# Archaeal genomes linked to industrial wastewater and associated freshwater in South Africa

**DOI:** 10.1128/mra.01079-24

**Published:** 2025-03-25

**Authors:** J. P. Makumbi, O. K. Bezuidt, S. K. Leareng, T. P. Makhalanyane

**Affiliations:** 1 Department of Biochemistry, Genetics and Microbiology, DSI/NRF SARChI in Marine Microbiomics, microbiome@UP, University of Pretoria 56410, Pretoria, South Africa; 2 Centre for Epidemic Response and Innovation, School for Data Science and Computational Thinking, Stellenbosch University 26697, Stellenbosch, South Africa; 3 Department of Microbiology, Faculty of Science, Stellenbosch University 26697, Stellenbosch, South Africa; Portland State University, Portland, Oregon, USA

**Keywords:** archaea, wastewater

## Abstract

Archaea provide important ecosystem services including the degradation of contaminants. Here, we present archaeal genomes from understudied South African wastewater treatment plants (WWTPs) and associated rivers receiving industrial effluents. Functional analysis revealed key genes implicated in heavy metal degradation, offering a valuable resource for mechanistic studies on archaeal metabolism.

## ANNOUNCEMENT

Studies across different environments have highlighted the importance of archaea in ecosystem services, such as biogeochemical cycling and as bioindicators of pollution (1–4). Despite this ecological significance, archaeal genomic characteristics remain poorly understood, compared with other microorganisms, such as bacteria (5). Contributing to this knowledge deficit is the underrepresentation of metagenomic data sets from the Global South, including from several African countries (6, 7). Here, we use genome-resolved metagenomics to report on archaea from urban wastewater treatment plants (WWTPs) and downstream river waters in South Africa. These WWTPs are subject to industrial effluents (8, 9), which may substantially impact the surrounding water.

Influent and effluent samples were collected from WWTPs (*n* = 6) in Tshwane, South Africa, between February and May 2023. River water and sediment samples were collected 100–200 meters upstream and downstream of the effluent discharge point. Water samples were taken 15–20 cm below the surface, and 250 g of sediment was collected from the top 5 cm of the riverbed. One liter of each water sample was filtered through 0.2 µm polyethersulfone (PES) membrane filters (47 mm diameter) (Millipore, USA). DNA was extracted from PES filters and sediment samples using the DNeasy PowerSoil Pro kit (QIAGEN, USA) according to the manufacturer’s instructions. The metagenomic DNA was sequenced (2 × 150 bp, yielding 50 million reads per direction for each sample) on the Illumina NovaSeq platform (Admera Health Biopharma Services, USA). Library preparation was performed using a KAPA Hyper Prep kit with PCR (Roche, Switzerland) per the manufacturer’s recommendations. Library quality and quantity were assessed with Qubit 2.0 DNA HS Assay (ThermoFisher, USA), Tapestation High Sensitivity D1000 Assay (Agilent Technologies, USA), and QuantStudio 5 System (Applied Biosystems, USA). Illumina 8-nt dual-indices (Illumina, USA) were used for indexing and barcoding. Equimolar pooling of libraries was performed based on QC values prior to sequencing.

The reads were trimmed to remove adapters using Trimmomatic v0.39 (10) and assembled into contiguous segments using MEGAHIT v1.2.9 (11). The contigs were mapped to quality-filtered reads using BWA v0.7.17 (12), indexed with Samtools v1.9 (13), and binned into metagenome-assembled genomes (MAGs) using MetaBAT 2 v2.15 (14). MAG quality was assessed with CheckM2 v1.0.1 (15) and categorized according to the Genomic Standards Consortium criteria (16). The taxonomy of MAGs was assigned using the Genome Taxonomy Database toolkit (GTDB-Tk) v2.3.0 (17), while selected metal degradation genes were predicted with EggNOG-mapper v5.0 (18) using the DIAMOND (v2.0.15) search mode. Default parameters were used for all software unless otherwise specified.

We recovered 19 archaeal MAGs, comprising 13 medium- and 6 high-quality genomes (Table 1). These MAGs were classified into the Nanoarchaeota (9), Methanobacteriota (5), Halobacteriota (3), Micrarchaeota (1), and Thermoproteota (1) phyla. Notably, the majority of these archaeal genomes (11/19) were recovered from effluents at WWTPs treating industrial wastewater. Metal degradation and transport genes (*ars*B, *ars*C, *cop*A, *cop*Z, *czc*D, and *feo*B) were detected in archaea from effluents and river sediments across all phyla, except Methanobacteriota. Taken together, these results highlight the potential of these archaeal taxa as bioindicators of pollution in contaminated environments.

**TABLE 1:** Sample details, genomic characteristics, and assembly statistics of the archaeal genomes

Genome ID	Sample type	Raw reads accession number	Genome accession number	Reads per MAG	Genome completeness (%)	Contamination (%)	Quality	Contig N50	Genome size (Mb)	GC content (%)	Phylum	Genus	Species	Metal degradation genes
20082D-13–12_S91_L002.bin.128	WWTP Effluent	SRR32414324	SAMN43934621	266,053	100	0.16	High	65662	1.3	33	Nanoarchaeota	UBA9642	Unclassified	copZ, copA
20082D-13–12_S91_L002.bin.161	WWTP Effluent	SRR32414324	SAMN43934623	410,393	92.65	0.81	High	291525	1.5	33	Nanoarchaeota	Unclassified	Unclassified	None detected
20082D-13–21_S46_L001.bin.92	WWTP Influent	SRR32414320	SAMN43934634	624,065	97.63	0.13	High	24476	1.6	31	Methanobacteriota	Methanobrevibacter_A	Methanobrevibacter A smithii	None detected
20082D-13–41_S107_L002.bin.19	River sediment	SRR32414317	SAMN43934640	1,255,410	93.81	3.15	High	37432	2.7	53	Halobacteriota	Methanothrix	Methanothrix sp002505805	arsB
20082D-13–45_S60_L001.bin.36	River sediment	SRR32414316	SAMN43934641	381,913	99.9	0.94	High	25910	1.6	31	Methanobacteriota	Methanobrevibacter_A	Methanobrevibacter A smithii	None detected
20082D-13–46_S61_L001.bin.104	River sediment	SRR32414315	SAMN43934642	362,783	94.18	0.52	High	12421	1.7	37	Methanobacteriota	Methanobacterium_A	Unclassified	None detected
20082D-13–12_S91_L002.bin.1	WWTP Effluent	SRR32414324	SAMN43934619	243,501	75.63	0.81	Medium	8614	1.9	52	Halobacteriota	Methanothrix	Unclassified	None detected
20082D-13–12_S91_L002.bin.105	WWTP Effluent	SRR32414324	SAMN43934620	569798	93.35	7.36	Medium	105742	0.95	31	Nanoarchaeota	MWBV01	Unclassified	None detected
20082D-13–12_S91_L002.bin.134	WWTP Effluent	SRR32414324	SAMN43934622	179,671	89.08	0.59	Medium	29305	0.9	56	Micrarchaeota	Unclassified	Unclassified	None detected
20082D-13–12_S91_L002.bin.40	WWTP Effluent	SRR32414324	SAMN43934624	244296	89.68	3.28	Medium	32553	1.2	37	Nanoarchaeota	Unclassified	Unclassified	copA
20082D-13–12_S91_L002.bin.42	WWTP Effluent	SRR32414324	SAMN43934625	439,782	91.8	5.46	Medium	13685	1.5	35	Nanoarchaeota	UBA9642	Unclassified	copZ, copA
20082D-13–12_S91_L002.bin.78	WWTP Effluent	SRR32414324	SAMN43934626	1506328	75.68	1.66	Medium	56684	1.1	31	Nanoarchaeota	Unclassified	Unclassified	None detected
20082D-13–12_S91_L002.bin.86	WWTP Effluent	SRR32414324	SAMN43934627	657,746	87.06	0.37	Medium	11367	1.1	37	Nanoarchaeota	UBA9642	Unclassified	None detected
20082D-13–14_S92_L002.bin.103	WWTP Influent	SRR32414323	SAMN43934628	288343	81.19	0.09	Medium	8571	1.6	32	Methanobacteriota	Methanobrevibacter_A	Methanobrevibacter A smithii	None detected
20082D-13–17_S95_L002.bin.89	WWTP Influent	SRR32414322	SAMN43934629	147,563	53.15	0.98	Medium	8171	0.6	33	Methanobacteriota	Methanobrevibacter_A	Methanobrevibacter A smithii	None detected
20082D-13–18_S96_L002.bin.89	WWTP Effluent	SRR32414321	SAMN43934631	338,242	89.14	0.68	Medium	163722	0.8	36	Nanoarchaeota	SICU01	Unclassified	copA, arsC
20082D-13–22_S98_L002.bin.129	WWTP Effluent	SRR32414319	SAMN43934635	248,424	87.42	4.56	Medium	15334	1.2	33	Nanoarchaeota	SICU01	Unclassified	copA
20082D-13–40_S56_L001.bin.26	River sediment	SRR32414318	SAMN43934638	371,320	61.63	1.58	Medium	6063	1.4	36	Thermoproteota	TA-21	Unclassified	czcD
20082D-13–41_S107_L002.bin.110	River sediment	SRR32414317	SAMN43934639	196,077	53.27	0.1	Medium	62351	1.2	51	Halobacteriota	UBA9949	Unclassified	arsC, feoB

## Data Availability

The genome assemblies for the MAGs, along with the raw reads, have been deposited in NCBI GenBank (BioSample) and the Sequence Read Archive (SRA) under BioProject PRJNA1165497 with the corresponding accession numbers listed in Table 1.
